# Editorial: Evaluating imaging modalities in the diagnosis of infective endocarditis

**DOI:** 10.3389/fcvm.2026.1761487

**Published:** 2026-01-15

**Authors:** Francesco Dondi, Domenico Albano, Giorgio Treglia

**Affiliations:** 1 Nuclear Medicine, Università Degli Studi di Brescia and ASST Spedali Civili di Brescia, Brescia, Italy; 2 Faculty of Biomedical Sciences, Università Della Svizzera Italiana, Lugano, Switzerland; 3 Faculty of Biology and Medicine, University of Lausanne, Lausanne, Switzerland; 4 Division of Medical Education and Research, Ente Ospedaliero Cantonale, Bellinzona, Switzerland

**Keywords:** cardiac valve, computed tomograghy, echocardiography, endocarditis, TAVI (transcatheter aortic valve implantation)

Infective endocarditis (IE) is a disease that, although rare, can lead to severe complications with high morbidity and mortality. This makes it essential to use and further develop diagnostic tools capable of providing an early and accurate diagnosis. This topic includes seven articles highlighting how different imaging approaches can aid in the detection of IE and in the assessment of disease extent and severity, through a series of case reports and a pictorial review.

Huang et al. reported an interesting case of a male patient on long-term hemodialysis whoe experienced an upper respiratory tract infection caused by Type I parainfluenza virus with subsequent progression to pneumonia. During hospitalization, auscultation revealed atypical diastolic “tumor plop” sound typical of atrial myxoma and further investigation with echocardiography reported an atrial mass measuring 51 × 33 × 32 mm, oscillating between the left atrium and ventricle. Subsequent blood cultures confirmed Rothia dentocariosa's bloodstream infection and the patient experienced an embolic stroke due to the detachment of the cardiac mass. All together, clinical findings supported the diagnosis of IE. Surgical removal of the mass and antibiotic therapy led to improvement of patient's clinical status.

Mao et al. published a case of a male with persistent fever after allogeneic hematopoietic stem cell transplantation (allo-HSCT) related to the presence of Volvariella volvacea infection. He was evaluated with transthoracic echocardiography reporting the presence of a left atrial vegetation of 4 × 1 cm and enhanced computed tomography (CT) revealed the presence of multiorgan septic emboli. Subsequent [18F]fluorodesoxyglucose positron emission tomography/CT confirmed the presence of left atrial vegetation spread via the pulmonary vein from a right lung infectious lesion. Urgent vegetation resection and intravenous antifungal treatment were performed and at follow-up the patients had complete improvement of conditions.

Sacra et al. presented a case of woman that underwent surgical aortic valve replacements (SAVR) for severe aortic regurgitation and membranous ventricular septal defect due to the presence of IE. The patient had also a diagnosis of double-chambered right ventricle. Ten years after the first valve implantation, she underwent a valve replacement again related to the presence of IE. A third SAVR was performed four years later after the development of Bordetella hinzii IE. Five years later, two-dimensional transesophageal echocardiography (TEE) revealed moderate-to-severe paravalvular leak (PVL) regurgitation near the right coronary cusp, subsequently corrected with trascatheter PVL closure guided by TEE/angio-fluoroscopic via the right femoral artery. Following imaging confirmed effective PVL closure with a trace-mild residual leak.

Li et al. proposed a case of a woman hospitalized for worsening weakness and cardiac tiredness with a negative cardiac color Doppler ultrasound examination. After the appearance of unconsciousness that required proper treatment, an emergency echocardiogram revealed the presence of an irregular and isoechoic mass attachment in the anterior leaflet of mitral valve (45 × 27 mm) and an ejection fraction of 30%. Therapy with extracorporeal membrane oxygen, fluid infusion, vasoactive agents and antibiotics were therefore started. After admission on another hospital, echocardiogram confirmed the neoplasm in the front valve of the mitral valve, however with smaller dimension (15 × 12 mm). Ultrasound of lower limbs revealed venous thrombosis in the right lower limb and CT underlined multiple cerebral and splenic infarction. The final diagnoses were IE, acute heart failure, cardiogenic shock, splenic infarct, cerebral infarction and necrosis of both lower limbs, nose and fingers of both hands related to Streptococcus mitis infection. Multidisciplinary team assessment underlined that risks of surgery were extremely high and the patient died on the same day after discharge.

Zheng et al. described the case of a woman with a 7-months history of fever and persistent chest discomfort. Initial tests showed leukopenia, anemia and elevated inflammatory markers. Transthoracic echocardiogram (TTE) demonstrated numerous echogenic vegetations affixed to the anterior mitral valve, including one particularly prominent of 17 × 11 mm, and severe mitral insufficiency strongly suggesting fungal IE. CT scanning and abdominal ecography resulted negative for embolic lesions. Despite refusing TEE, she underwent urgent valve replacement that revealed the presence of additional small vegetations and a perforation on the aortic valve which required mechanical mitral and aortic valves were implanted. Blood cultures and intraoperative biopsies confirmed the presence of Candida guilliermondii infection, treated therefore with fluconazole therapy and, after completion, the patient remained asymptomatic and fully recovered at subsequent follow-up.

Cabrucci et al. published a case of a woman who underwent transfemoral transcatheter aortic valve implantation (TAVI) that presented with fatigue, dyspnea and fever. Blood tests showed leukocytosis and elevated procalcitonin while blood cultures grew Staphylococcus aureus. TTE and TEE revealed the presence of large vegetations on the prosthetic valve, severe non-structural dysfunction, and high transvalvular gradients. Subsequent angio-CT scanning revealed thrombosis between the left coronary and non-coronary cusps of the prosthesis and splenic embolization. Antibiotic therapy was started but her condition deteriorated, prompting reconsideration of surgery despite extremely high surgical risk. Prosthetic valves were removed and replacement of the ascending aorta and valve were performed. The patient recovered well and remained event-free at 15-month follow-up.

Lastly, Montarello et al. proposed a pictorial review of seven cases with the aim to explore the value of systematic cardiac CT for the diagnosis and management of IE, in particular when echocardiography is inconclusive. CT identified key findings such as vegetations on native and prosthetic valves, pseudoaneurysms, abscesses, fistulae, leaflet perforations, and septic emboli, many of which were missed or insufficiently characterized by TTE or TEE. CT proved particularly advantageous in prosthetic valve IE, where artifacts often hinder echocardiographic assessment, and in defining the extent of paravalvular disease. The authors moreover highlights how modern IE guidelines increasingly integrate multimodality imaging. In this setting, while echocardiography remains first-line for detecting vegetations and assessing valve function, CT offers superior visualization of paravalvular structures, extracardiac complications, and coronary anatomy, contributing to surgical planning and risk assessment. Limitations of this diagnostic procedure include radiation, contrast use, artefacts, and reduced utility for small vegetations. Overall, CT was endorsed as a complementary, sometimes essential, tool for achieving a definite IE diagnosis and guiding timely intervention.

In conclusion, this Research Topic comprises several studies highlighting the potential applications of various imaging modalities in the diagnosis and management of IE, thereby supporting a more personalized approach to patient care.

